# Dynamically Tunable and Multifunctional Polarization Beam Splitters Based on Graphene Metasurfaces

**DOI:** 10.3390/nano12173022

**Published:** 2022-08-31

**Authors:** Gongli Xiao, Sitong Zhou, Hongyan Yang, Zhixiong Lin, Haiou Li, Xingpeng Liu, Zanhui Chen, Tangyou Sun, Peihua Wangyang, Jianqing Li

**Affiliations:** 1 Guangxi Key Laboratory of Precision Navigation Technology and Application, Guilin University of Electronic Technology, Guilin 541004, China; 2 Guangxi Key Laboratory of Optoelectronic Information Processing, School of Optoelectronic Engineering, Guilin University of Electronic Technology, Guilin 541004, China; 3 Guangdong-Hong Kong-Macao Joint Laboratory for Intelligent Micro-Nano Optoelectronic Technology, Macau University of Science and Technology, Macau 999078, China

**Keywords:** plasmon-induced transparency, graphene, polarization beam splitter, polarization-sensitive, metamaterials

## Abstract

Based on coupled-mode theory (CMT) and the finite-difference time-domain (FDTD) approach, we propose a graphene metasurface-based and multifunctional polarization beam splitter that is dynamically tunable. The structure, comprising two graphene strips at the top and bottom and four triangular graphene blocks in the center layer, can achieve triple plasma-induced transparency (PIT). In a single polarization state, the computational results reveal that synchronous or asynchronous six-mode electro-optical switching modulation may be performed by modifying the Fermi levels of graphene, with a maximum modulation degree of amplitude (MDA) of 97.6% at 5.148 THz. In addition, by varying the polarization angle, a polarization-sensitive, tunable polarization beam splitter (PBS) with an extinction ratio and insertion loss of 19.6 dB and 0.35 dB at 6.143 THz, respectively, and a frequency modulation degree of 25.2% was realized. Combining PIT with polarization sensitivity provides a viable platform and concept for developing graphene metasurface-based multifunctional and tunable polarization devices.

## 1. Introduction

Polarization, along with amplitude and phase, is an essential property of electromagnetic wave propagation. A polarization beam splitter (PBS) is a crucial polarization modulation device that can separate two orthogonal polarization modes in electromagnetic waves and can be used in single-polarization devices within various parts of an integrated system so that subsequent chip signal processing can be performed independently. Consequently, it has significant applications in optical storage, optical communication, and integrated optical circuits [1,2,3], and has become a popular research topic in recent years. Electromagnetically induced transparency (EIT) [4,5] is a unique optical phenomenon in which light is destructively interfered with between atomic energy levels, making EIT applicable to the fields of sensing [6,7] and slow light [8,9]. However, EIT requires rigorous experimental conditions, severely restricting its optical application. Thus, EIT-like optical phenomena based on the theory of surface plasmon polaritons (SPPs) emerged: plasmon-induced transparency (PIT), which is generated by the coupling excitation between bright and dark modes [10]. In contrast to EIT, PIT requires less stringent experimental conditions, leading to its extensive use in optical research.

Many scientists prefer graphene because it exhibits less major losses in metamaterials than conventional metallic materials. Metamaterials made of graphene also benefit from dynamic modulation, which modifies the graphene’s Fermi levels by varying the externally supplied bias voltage to induce various visual phenomena. In optical devices, including electro-optical switches [11,12,13,14], optical modulators [15,16], optical absorbers [17,18,19,20,21], slow light devices [22,23], and various sensors [24,25,26], graphene is widely used in these applications. Luo et al., reported a review on the research progress of 2D material-based absorbing materials in visible and near-infrared light, focusing on the preparation of several typical ideal absorbing materials, such as narrowband, dualband, and broadband, and the related physical mechanisms [21]. This can help researchers to better understand 2D material-based absorbing materials in the visible and near-infrared regions. In addition, the structure designed by Luo et al., that couples a silicon array in a two-layer graphene waveguide exploits the dynamically tunable properties of graphene [17]. By manipulating the Fermi energy levels of two graphene layers, two coherent absorption peaks can move over a wide spectrum range, and the designed coherent perfect absorber (CPA) can also be changed from dualband to narrowband CPA. Their proposed graphene/hexagonal boron nitride van der Waals hybrid structure can maintain good absorption performance in large angular incidences, and its biggest advantage is that its graphene does not need to be patterned, which largely reduces the fabrication difficulty in the actual production process [20], all of which provides good ideas for the design of graphene-based optoelectronic devices.

However, the majority of these devices only work in one polarization state. PBSs have become a popular area of research because integrated systems require a polarization control mechanism to maintain a single polarization state across the system. Due to their low process needs and straightforward design, most PBSs are currently constructed using the directional coupling design approach. For instance, Tian et al., developed a portable ultra-wideband PBS [27], while Bai et al., created an ultra-short plasmonic polarization beam splitter–rotator employing a bent directional coupler [28]. However, because the operating band of this PBS is fixed, altering the device’s structural specifications is required, which raises the cost of production. Consequently, due to graphene’s distinctive dynamically adjustable features, it is frequently used in the research of PBSs. By altering the chemical potential of the graphene, these PBSs can be dynamically tailored to the operational bandwidth or operating frequency point. As some PBSs only accomplish very straightforward tasks, there are times when it is required to include auxiliary units in an integrated system, which increases the size of the integrated system as a whole. As a result, integrated optical circuits have a lot of room for multifunctional terahertz devices. The creation of a graphene-based electrothermally tunable subsurface array by Wang et al. [29] that can simultaneously perform beam splitting and filtering tasks has important ramifications for integrating terahertz systems. In addition, Zhang et al., discovered in their investigation of triple PIT that when the polarization angle was adjusted by 90°, the PIT phenomena that the structure had initially produced vanished [30]. In polarization devices, a variety of uses for this feature are anticipated. It provides yet another fresh concept about the polarization of metamaterials with multiple benefits. Currently, chemical vapor deposition (CVD) technology is generally used to achieve the preparation of graphene [31]. Ni rods were prepared on the surface of the substrate using electron-beam lithography (EBL). Then, graphene was grown on the Ni rods through rapid heating, using a mixture of methane and hydrogen gas. During subsequent rapid cooling, the nickel rod structure was transformed into graphene bands accompanied by the volatilization of nickel [32]. Triangular graphene patterns can be grown on cube-textured (100) copper foils through CVD [33] and then graphene can be transferred to silicon substrates and dielectric films by transfer technology [34], but this will cause unavoidable contamination and damage to the graphene, to some extent, during the transfer process. Cric et al., reported the recent research progress on multilayer MMS, which supports the structure of multilayer dielectric stacking of the present design from the theoretical level [35].

This work proposes a triple-layer metamaterial made up of two graphene strips and four graphene blocks. Finite-difference time-domain (FDTD) simulations were performed [36], and coupled-mode theory (CMT) was utilized for theoretical analysis [37]. In the single polarization state, changing the Fermi levels of graphene caused a noticeable phase variation in the transmission spectra, allowing for the realization of a six-mode electro-optical switch with amplitude modulation of up to 97.6 %. Furthermore, it was discovered that the structure was polarization-sensitive in the dual-polarization state and that it could generate a tunable PBS with a polarization extinction ratio (PER) of up to 19.6 dB, a minimum insertion loss (IL) of 0.36 dB, and a modulation degree of frequency (MDF) of up to 25.2%. Then, we explored the mechanism of metamaterial polarization sensitivity. In this study, the multifunctional PBS can be employed as a switching unit to choose a frequency, or modulate a transmission path in an integrated system in a single polarization state. In the dual-polarization form, it can be used as an integrated system’s primary polarization modulation component to separate two orthogonal polarization beams into a single polarization device. The present work is the first to use PIT to transmit and reflect the two polarization states separately, thus realizing beam splitting of the two orthogonal polarization states. It will be quite promising in the field of multifunctional integrated chips.

## 2. Materials and Methods

**Figure 1:**
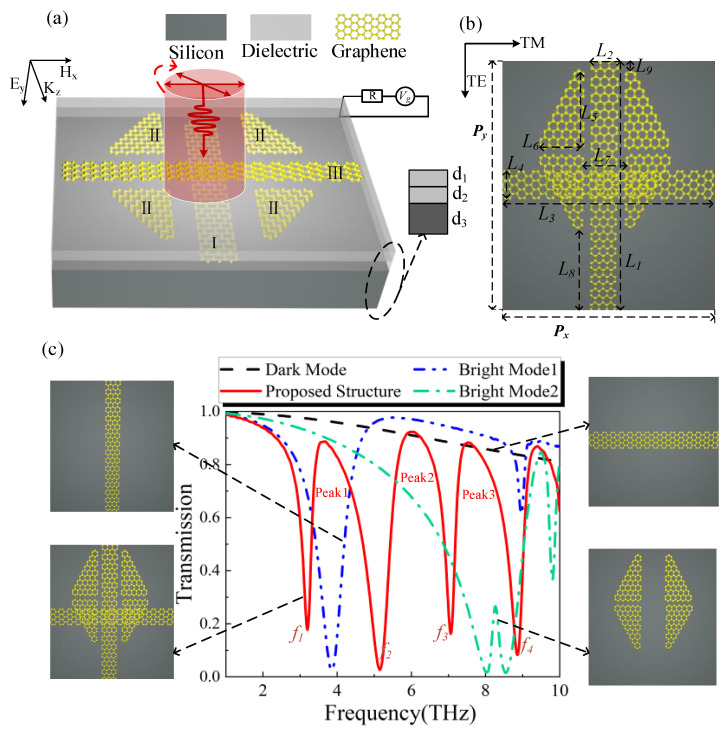
(**a**) Schematic diagram of the periodic 3D unit structure of the metamaterials. (**b**) A 2D top view of the unit structure. (**c**) Transmission spectra of different structures under *y*-polarized light irradiation when the Fermi levels being set to 1 eV.

Figure 1a shows the periodic unit of the three-layer graphene metamaterial structure. The lower substrate is silicon with $d_{3}=0.7\;\mu m$, the top two layers are dielectric films with $d_{1}=d_{2}=20\;\text{nm}$, and the dielectric permittivity is 1.4. Ⅰ, Ⅱ, and Ⅲ denote the layer where the graphene structure is located. Chen at al. demonstrated a bottom-up approach to fabricate scalable nanostructured metamaterials via spinodal decomposition, and good experimental results were achieved [38]. Xia et al., proposed a structure based on the mutual coupling of upper and lower nanoribbons to produce PIT and obtained good results. These provide reliable results for the experimental and theoretical aspects of the present work [39]. The external power section is the bias voltage source and resistance used to control the Fermi levels of graphene. In practice, the resistance and transmission line may cause losses, which are neglected here. In addition, if the Fermi levels of graphene are set too high, it will deviate from the scope described by Dirac [40] and become diffusive transport; therefore, the Fermi levels are usually placed below 1 eV.

The top view structural unit of the graphene metamaterials is shown in Figure 1b, and the geometric parameters are as follows: $L_{1}$ = $L_{3}$ = 10 μm, $L_{2}$ = $L_{4}$ = 0.6 μm, $L_{5}$ = $L_{6}$ = 3.3 μm, $L_{7}$ = 0.6 μm, $L_{8}$ = 2.7 μm, $L_{9}$ = 0.3 μm, and $P_{x}$ = $P_{y}$ = 11 μm. Firstly, we start the simulation with *y*-polarized light, and transmission spectra as shown in Figure 1c. The blue line in Figure 1c depicts Bright Mode 1, a bright mode that can be directly excited at 3.851 THz when the graphene strip in the *y*-direction is present alone. The graphene strip in the *x*-direction is not instantly activated by the incident light when it exists independently. Instead, it belongs to a dark mode, as indicated by the black line in Figure 1c.

**Figure 2:**
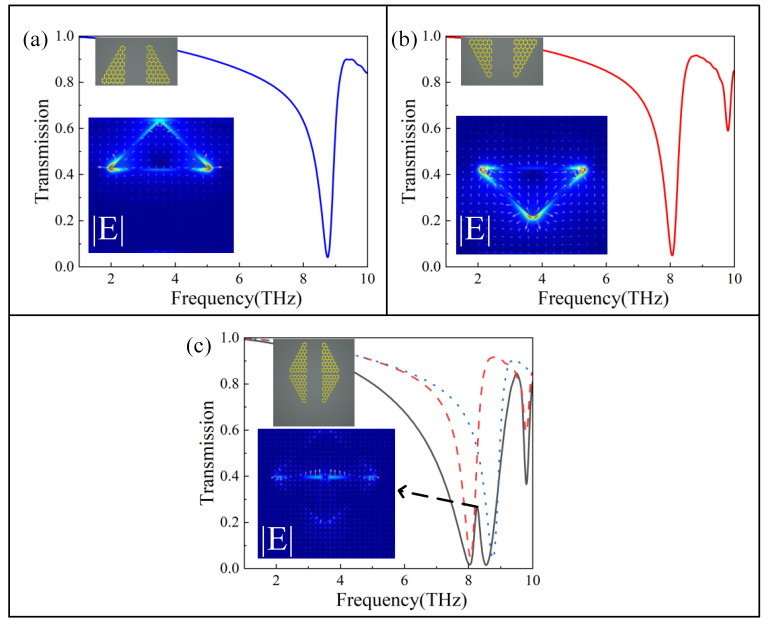
Transmission spectra of graphene blocks. The blue solid and dotted lines are the transmission spectrum of the upper graphene block, and the red solid and dotted lines are the transmission spectrum of the lower graphene block. (**a**) The two graphene blocks in the upper part. (**b**) The two graphene blocks in the lower part. (**c**) Four graphene blocks.

As shown by the green line in Figure 1c, when the four graphene blocks in the intermediate layer are present separately, two transmission valleys appear at 8.034 THz and 8.539 THz. As the transparency peak of 8.27 THz is very weak, it makes it look like a bright mode, but in fact these are two bright modes produced by the direct excitation of incident light at different frequencies, and we will explore the mechanism of this phenomenon in detail below. As shown in Figure 2a,b, the transmission spectra of the two graphene blocks above and the two graphene blocks below when they are alone, respectively, can both be directly excited by the incident light at different frequency points. As shown in Figure 2c, when four graphene blocks are placed together, their transmission spectrum has a weak transparency peak of 8.27 THz, which is an electromagnetically induced transparency phenomenon caused by the mutual coupling of two bright modes, generated by the interference of different excitation paths with each other, and this work has been investigated in depth by many researchers [5,41]. According to the field distribution maps in Figure 2a,b, we find that in-plane dipole resonances with different orientations appear, and the transparency peaks are mainly due to their mutual interferences. However, its EIT is relatively weak, so in essence, the structure of four graphene blocks after being combined together can be directly excited twice by incident light at different frequencies, producing two bright modes. It can be seen that the two transmission valleys of the green line in Figure 1c are two bright modes excited directly by the incident light at different frequencies.

**Figure 3:**
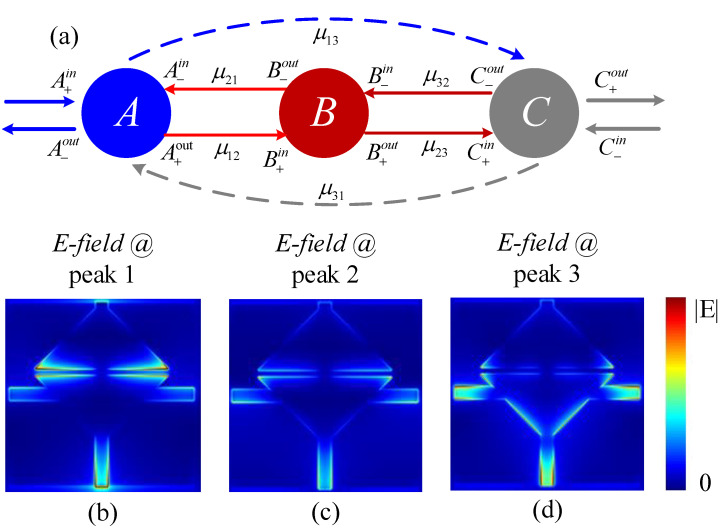
(**a**) Schematic diagram of CMT, *A* corresponds to Bright Mode 1, *B* corresponds to Bright Mode 2, and *C* corresponds to Dark Mode, here $\mu_{mn}=10^{4}cm^{-2}v^{-1}s^{-1}$. (**b**–**d**) The corresponding electric field distribution at three transmission peaks.

As a result, incoming light can stimulate it twice, making it a bright mode (Bright Mode 2). Triple PIT is produced at Peak 1, Peak 2, and Peak 3 when the three structures are combined. Furthermore, when the three-layer graphene structure is present, Bright Mode 1 influences the interstitial coupling between the graphene blocks, which results in some deviation of Peak 2 from the resonance point at 8.034 THz and transmission spectra of the entire structure, as shown by the red line in Figure 1c. The schematic diagram is similar to that in Figure 3a, based on the CMT, to analyze the PIT phenomenon. Each mode is represented by a spherical resonant cavity, with resonant cavity *A* representing Bright Mode 1, resonant cavity *B* representing Bright Mode 2, and resonant cavity *C* representing Dark Mode. Figure 3b,d show the corresponding electric field distributions at the three transmission peaks and Bright Mode 2, which the incident light at Peak 1 cannot directly activate. However, because Bright Mode 1 is present, two bright modes are coupled at Peak 1, leading to the first PIT emergence. As can be seen in Figure 3c,d, Bright Mode 2 couples and excites the Dark Mode twice, resulting in the second and third PIT at Peaks 2 and 3, respectively. Bright Mode 1 is constantly participating in energy transfer.

Based on the schematic diagram, the relationship between resonant cavities *A*, *B*, and *C* can be expressed by the following equation [30].

(1) $$\left(\begin{array}{ccc} \gamma_{1} & -i\mu_{12} & -i\mu_{13}\\ -i\mu_{21} & \gamma_{2} & -i\mu_{23}\\ -i\mu_{31} & -i\mu_{32} & \gamma_{3} \end{array}\right)\cdot \left(\begin{array}{c} a\\ b\\ c \end{array}\right)=\left(\begin{array}{ccc} -\gamma_{o1}^{½} & 0 & 0\\ 0 & -\gamma_{o2}^{½} & 0\\ 0 & 0 & -\gamma_{o3}^{½} \end{array}\right)\cdot \left(\begin{array}{c} A_{+}^{in}+A_{-}^{in}\\ B_{+}^{in}+B_{-}^{in}\\ C_{+}^{in}+C_{-}^{in} \end{array}\right)$$

where $\mu_{mn}\left(m,n=1,2,3,m\neq n\right)$ is the mutual coupling coefficient between resonant cavities *A*, *B*, and *C*, and is related to the thickness of the dielectric films $d_{1},d_{2}$. *A*, *B*, and *C* are the amplitudes of the three modes, “in/out” are the input or output waves, and “±” is the direction of wave propagation. $\gamma_{n}=\left(i\omega -i\omega_{n}-\gamma_{in}-\gamma_{on}\right)$(n=1,2,3), ω is the angular frequency of the incident wave, and $w_{n}\left(n=1,2,3\right)$ is the frequency of the n-th resonance angle. $\gamma_{in}$ and $\gamma_{on}$ are internal losses and external losses.

$\gamma_{in}=½\cdot \omega_{n}/Q_{in}$, $\gamma_{on}=\omega_{n}/2Q_{tn}-\omega_{n}/2Q_{in}$, Qtn=fΔf, Qin=Re(β/K0), where f and Δf are the resonant frequency of the transmission spectra and its corresponding full-width half-maximum (FWHM), respectively. β indicates the propagation constant. As the top layer is different from the bottom medium, the propagation constant of the middle layer is expressed by the following equation:

(2) β=ςsi−(2ςsiση0)2⋅K0 ,

The propagation constant between the top and bottom layers is expressed by the following equation:

(3) ςsiβ2−ςsi⋅K02+ςDβ2−ςD⋅K02=−iσως0,

where $K_{0},\eta_{0},\varsigma_{si},\varsigma_{D},\varsigma_{0},\sigma$ represent wave vector, intrinsic impedance, the relative permittivity of silicon, dielectric film, and air, and the conductivity of graphene, respectively, where the conductivity of graphene is obtained by Kubo [42].

The plane wave enters from resonant cavity *A* and emits from resonant cavity *C*. Therefore, it can be understood as follows:

(4) $$C_{-}^{in}=0,$$

The expression for the energy input between *A*, *B*, and *C* is expressed as:

(5) $$A_{-}^{in}=B_{-}^{out}\cdot \exp\left(i\alpha_{1}\right),B_{+}^{in}=A_{+}^{out}\cdot \exp\left(i\alpha_{1}\right),$$

(6) $$B_{-}^{in}=C_{-}^{out}\cdot \exp\left(i\alpha_{2}\right),C_{+}^{in}=B_{+}^{out}\cdot \exp\left(i\alpha_{2}\right),$$

The expression for the energy output between *A*, *B*, and *C* is expressed as:

(7) $$A_{-}^{out}=A_{-}^{in}-\gamma_{o1}^{½}\cdot a,A_{+}^{out}=A_{+}^{in}-\gamma_{o1}^{½}\cdot a,$$

(8) $$B_{-}^{out}=B_{-}^{in}-\gamma_{o2}^{½}\cdot b,B_{+}^{out}=B_{+}^{in}-\gamma_{o2}^{½}\cdot b,$$

(9) $$C_{-}^{out}=C_{-}^{in}-\gamma_{o3}^{½}\cdot c,C_{+}^{out}=C_{+}^{in}-\gamma_{o3}^{½}\cdot c,$$

where $\alpha_{1},\alpha_{2}$ are the phase differences between *A* and *B*, and *B* and *C*, respectively. According to the above equation, the transmission coefficient *t* is expressed as:

(10) t=C+outA+in=exp(iα1+iα2)−exp(iα1+iα2)⋅γo1½⋅τa−exp(iα2)⋅γo2½⋅τb−γo3½⋅τc,

Finally, after the above series of derivations, the transmittance is expressed as:

(11) $$T={|t|}^{2},$$

## 3. Results and Discussion

The Fermi energy level of graphene can be modulated by changing the surface conductivity σ. When *E_f_* >> K_B_T, surface conductivity can be simplified as [43,44].

(12) $$\sigma (\omega )=\frac{e^{2}E_{f}}{\pi \hbar^{2}}\cdot \frac{i}{\omega +1/\tau },$$

where K_B_, T, and *E_f_*, are the Boltzmann constant, temperature, and Fermi levels, respectively. τ is relaxation time, where $\tau =\mu E_{f}/\left(e\upsilon_{F}^{2}\right)$, and $v_{F}$ is the Fermi velocity ($v_{F}=10^{6}\;m\cdot s^{-1}$).

### 3.1. Incidence of y-Polarized Light

**Figure 4:**
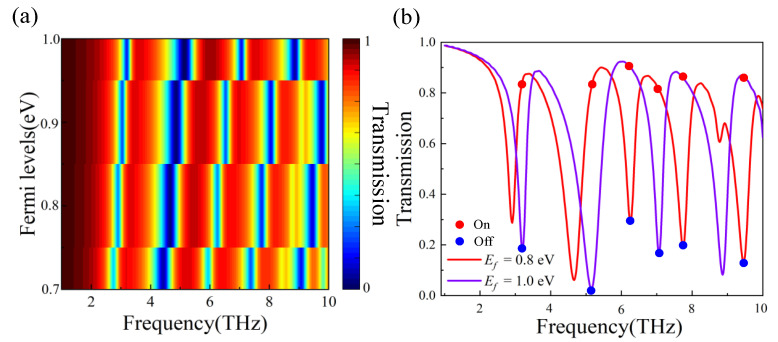
(**a**) Evolution of transmission spectra at different Fermi levels. (**b**) Comparison of the transmission spectra at $E_{f}=0.8\mathrm{eV}$, and $E_{f}=1.0\mathrm{eV}$.

Firstly, we analyze the results in the *y*-polarized state, as depicted in Figure 1b’s structural diagram. Figure 4a shows the intermittent evolution of the transmission spectra, as the Fermi levels were tuned from 0.7 to 1.0 eV. We can see that there is a significant phase difference between the peaks of the transmission spectra at different Fermi levels. Therefore, this provides a reliable result for dynamically tunable optical switching.

As shown in Figure 4b, a comparison of the transmission spectra at the Fermi levels tuned to 0.8 and 1.0 eV intuitively shows the mechanism of the electro-optical switch implementation, as described earlier. When $E_{f}=1.0\;\mathrm{eV}$, the state of the electro-optical switch is “off” at 3.201 THz, 5.148 THz, and 7.062 THz, and when $E_{f}=0.8\;\mathrm{eV}$, these states become “on”. On the other hand, when $E_{f}=1.0\;\mathrm{eV}$ the state is “on” at 6.248 THz, 7.745 THz, and 9.458 THz, and then at $E_{f}=0.8\;\mathrm{eV}$, the state is “off”. These features enable the switch to be synchronously and asynchronously modulated on–on or on–off, simultaneously.

The performance of the electro-optical switch can be expressed by the modulation degree of amplitude (MDA) [30]:

(13) $$MDA=\frac{\left|A_{on}-A_{off}\right|}{A_{on}}\times 100\%,$$

where $A_{on}$, $A_{off}$ are the amplitude of the “on” and “off” states in the transmission spectra, respectively. Thus, the MDA of six frequency points are 80.1%, 97.6%, 67.8%, 80.2%, 80%, and 84.9%, respectively. The realization of dynamic synchronous or asynchronous modulation is of great importance when researching tunable electro-optical switches. This also provides a good result for this study as it shows that multifunctional PBSs in a single polarization state can be used in integrated systems for frequency selection.

### 3.2. Influence of Polarization Angle

**Figure 5:**
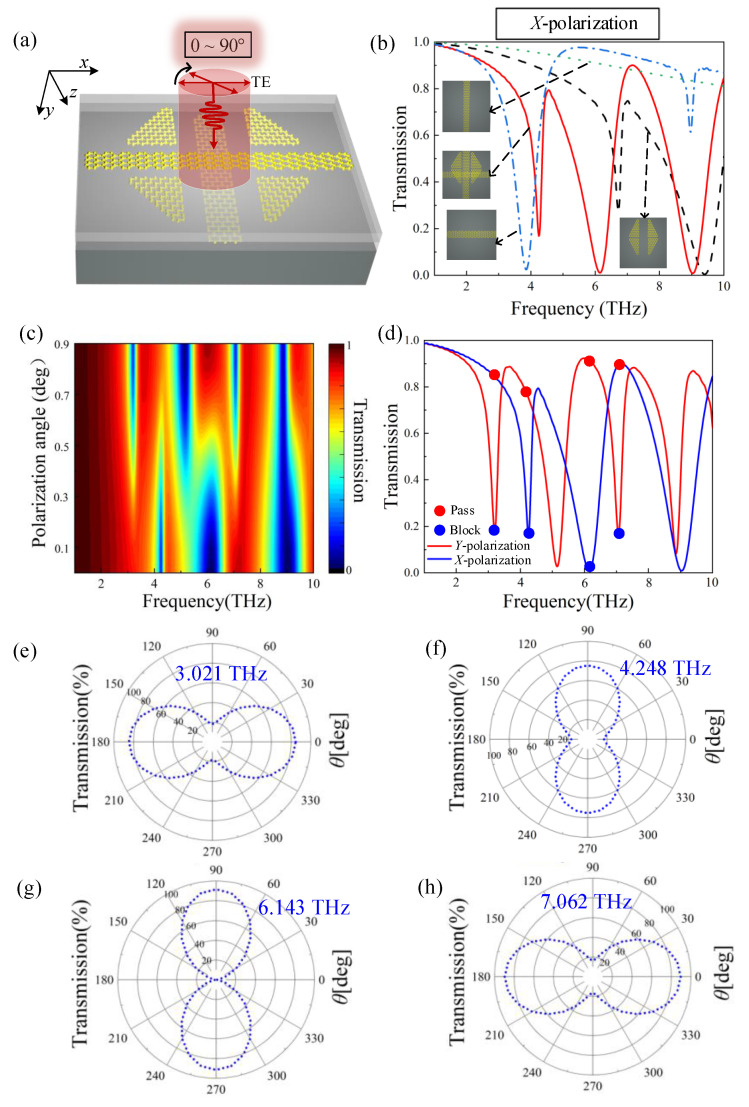
(**a**) Schematic diagram of the structure of the light polarization angle varying from 0° to 90°. (**b**) Transmission spectra of different structures under *x*-polarization. (**c**) Evolution of the transmission spectra with varying angles of polarization. (**d**) Transmission spectra of *x*-polarization and *y*-polarization. (**e**–**h**) Transmittance function with polarization angle as a variable.

Firstly, the polarization angle of the incident light is varied from 0° to 90°, as shown in Figure 5a, to investigate the sensitivity of the triple-layer graphene metamaterial structure to polarization. Figure 5c depicts the transmission spectra evolution with the polarization angle change. It is evident from this figure that the structure is susceptible to polarization at the four frequencies of 3.021 THz, 4.248 THz, 6.143 THz, and 7.062 THz. Consequently, this demonstrates that this optical switch can control the peak by adjusting the polarization angle and enables the use of polarization-sensitive properties to create polarization-modulated devices.

As shown in Figure 5b, we analyzed the cause of the polarization sensitivity. We discovered that coupling excitation between the three-layer graphene structure does not generate PIT in the *x*-polarized state. Due to the different pinch distances of the graphene blocks in the *x* and *y* directions, the distance between the two resonance points increases. Still, *x*-polarization does not couple with the Dark Mode to generate the PIT. These are the primary reasons for the polarization sensitivity of this structure. The polarization sensitivity we analyzed coincides with Zhang’s theory that the PIT of metamaterials is destroyed when the polarization angle changes [30].

The four frequency points’ specific modulation positions and implementation mechanism are denoted in Figure 5d, which illustrates the four-frequency polarizer’s operating principle. At 4.248 THz and 6.143 THz, the *y*-polarization is selected, and the *x*-polarization is filtered out. Instead, the *x*-polarization is filtered out at 3.201 THz, and the *y*-polarization is filtered out at 7.062 THz. These enable the realization of a multi-frequency polarizer. When combined with the polar coordinates in Figure 5e–h, the modulation effect of the four frequency points is readily apparent. The greater the modulation effect of the polarizer, the lower the transmittance at the center of the polar coordinates. The optimal performance can be found at 6.143 THz.

**Figure 6:**
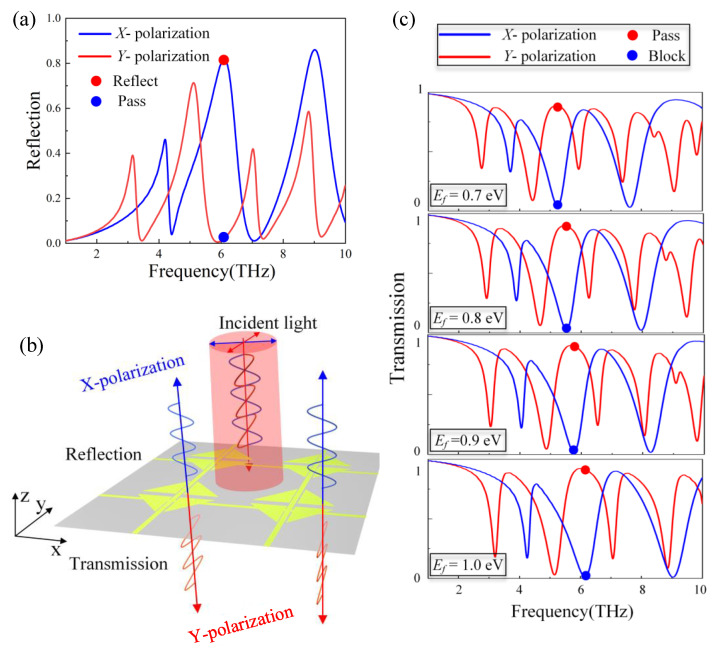
(**a**) Reflection spectrum of *x*- and *y*-polarized lights. (**b**) Operating schematic of PBS. (**c**) Frequency points of PBS operation at different Fermi energy levels of graphene.

As shown in Figure 6a, we plotted the polarizer’s reflection spectrum to investigate its function further. In both polarization states, the structure has some absorption at all four frequency points, which is due to the unique optical properties of graphene [45]. According to the formula for absorption A=1−T−R, as shown in Figure 5d and Figure 6a, the absorbances are 0.03, 0.4, 0.18, and 0.08 for *x*-polarization and 0.42, 0.04, 0.09, and 0.4 for *y*-polarization, respectively. As mentioned in some articles, the transmittance is considered to be in a normal operating state when it is greater than 0.8 [12]. Only at 6.143 THz, the results under two polarization states are the least affected by absorption, and both reflectance and transmittance are greater than 0.8, so it is in normal operation. The other three points are too affected by the absorption under a certain polarization to realize the polarization beam splitting function well, so they are not discussed here. At 6.143 THz, we observe that the filtered *x*-polarization wave is reflected with a reflectivity of up to 86% and almost no absorption, so we can assume it is entirely reflected back. At this frequency point, the orthogonal polarizations are spatially separated, creating an excellent polarization beam splitter. As shown in Figure 6b, the operating principle’s diagram of a polarization beam splitter, *x*-polarized light is reflected, and *y*-polarized light is transmitted directly. In addition, the performance of a PBS is typically determined by the polarization extinction ratio (PER) and insertion loss (IL), as shown in the following equations [28]:

(14) PER=10lg(TmaxTmin),

(15) $$IL=-10\mathrm{lg}\left(T_{\max}\right),$$

where $T_{\max}$ and $T_{\min}$ are the transmittance in the “open” and “closed” states, respectively. The PER at four frequency points is 6.7 dB, 6.5 dB, 19.6 dB and 7.5 dB. The IL are 0.7 dB, 1.23 dB, 0.36 dB and 0.46 dB, respectively. Next, as shown in Figure 6c, we adjusted the Fermi energy level of graphene and discovered that the transmission spectra under *x*- and *y*-polarization were synchronously blue-shifted.

This PBS can be dynamically modulated by changing the Fermi levels of graphene. The modulation degree of frequency is typically used to describe the frequency modulation effect, as shown in the following equation:

(16) $$MDF=\frac{\left|f_{\max}-f_{\min}\right|}{f_{\min}}\times 100\%$$

where $f_{\max}$ and $f_{\min}$ represent the operating frequencies at Fermi energy levels of 1 eV and 0.7 eV, respectively. The MDF of this PBS reached 25.2%. To demonstrate the performance of this metamaterial, the optimal parameters for each structure are listed in Table 1 for comparison.

## 4. Conclusions

In conclusion, we simulated a three-layer metamaterial structure of two graphene bands and four graphene blocks. Based on plasma-induced transparency, a six-mode dynamically tunable synchronous and asynchronous electro-optical switch was achieved by tuning the Fermi levels of graphene; its MDA is 80.1%, 97.6%, 67.8%, 80.2%, 80%, and 84.9 % at 3.021 THz, 5.148 THz, 6.248 THz, 7.062 THz, 7.745 THz, and 9.458 THz, respectively. Moreover, we explored the impact of polarization angle variation on graphene metamaterials. The results demonstrate that a change in the polarization angle disrupts the PIT formed by the structure, resulting in polarization sensitivity. Consequently, a polarization beam splitter was constructed with PER and IL values of 19.6 dB and 0.36 dB, respectively. In addition, based on the dynamic tunability of graphene, the operating frequency point of the polarization beam splitter is adjustable with an MDF up to 25.2%. This research lays the groundwork for the future construction of graphene-based multifunctional optoelectronic devices and configurable polarization beam splitters. The findings provide a theoretical basis for designing and implementing graphene-based, multifunctional, fully transmittable metasurface beam splitters for on-chip integration.

## Figures and Tables

**Table 1:** Performance comparison of different graphene structures.

Various Metamaterial	Modulated Frequency Points	Modulation Method	MDA/%	PER/dB	MDF/%
Triple-layer patterned graphene [44]	Four points	Synchronous	87.8, 77.7, 76.5, 74.7	9.15	
Triple-layer patterned graphene [16]	Two points	Synchronous	98, 68		
L-shape graphene [13]	Four points		97.3	11.34	
Single-layer patterned graphene [30]	Four points	Synchronous and asynchronous	77.7, 77.6, 75.4, 58.9	12.5	16.2
Structure of this article	Six points	Synchronous and asynchronous	97.6, 84.9, 80.2, 80.1, 80, 67.8	19.6	25.2

## Data Availability

The data that support the findings of this study are available from the corresponding author upon reasonable request.
